# RETRACTION: The Effects of Virtual Reality Technology on Reducing Pain in Wound Care: A Meta‐Analysis and Systematic Review

**DOI:** 10.1111/iwj.70550

**Published:** 2025-04-18

## Abstract

**RETRACTION:** HeZ.‐H. , YangH.‐M. , Dela RosaR. D. , De AlaM. B. , “The Effects of Virtual Reality Technology on Reducing Pain in Wound Care: A Meta‐Analysis and Systematic Review,” International Wound Journal 19, no. 7 (2022): 1810–1820, 10.1111/iwj.13785.35318806 PMC9615291

The above article, published online on 23 March 2022, in Wiley Online Library (http://onlinelibrary.wiley.com/), has been retracted by agreement between the journal Editor in Chief, Professor Keith Harding; and John Wiley & Sons Ltd. Following an investigation by the publisher, all parties have concluded that this article was accepted solely on the basis of a compromised peer review process. The editors have therefore decided to retract the article. The authors did not respond to our notice regarding the retraction.

**RETRACTION:** Z.‐H. He , H.‐M. Yang , R. D. Dela Rosa , M. B. De Ala , “The Effects of Virtual Reality Technology on Reducing Pain in Wound Care: A Meta‐Analysis and Systematic Review,” International Wound Journal 19, no. 7 (2022): 1810–1820, 10.1111/iwj.13785.35318806 PMC9615291

The above article, published online on 23 March 2022, in Wiley Online Library (http://onlinelibrary.wiley.com/), has been retracted by agreement between the journal Editor in Chief, Professor Keith Harding; and John Wiley & Sons Ltd. Following an investigation by the publisher, all parties have concluded that this article was accepted solely on the basis of a compromised peer review process. The editors have therefore decided to retract the article. The authors did not respond to our notice regarding the retraction.

